# In vitro and in vivo evaluation of protein quality of enzymatic treated feather meals

**DOI:** 10.1186/s40064-016-2626-2

**Published:** 2016-07-04

**Authors:** Warintorn Eaksuree, Akkharadet Prachayakitti, Tewa Upathanpreecha, Rutjawate Taharnklaew, Sunee Nitisinprasert, Suttipun Keawsompong

**Affiliations:** Department of Biotechnology, Faculty of Agro-Industry, Kasetsart University, Chatuchak, Bangkok, 10900 Thailand; Special Research Unit: Probiotic and Prebiotics for Health, Center for Advanced Studies for Agriculture and Food (CASAF), Institute for Advanced Studies, Kasetsart University, Chatuchak, Bangkok, 10900 Thailand; Research and Development Center of BETAGRO Group, Science Park, Klong Nueng, Klong Luang, Pathum Thani 12120 Thailand

**Keywords:** *Bacillus*, Feather meal, Keratinase, Broiler

## Abstract

Feeding trials were designed to evaluate the nutritive value of feather meal treated by K6 and K82 keratinase. There were five treatments in feather meal preparation: CFM (non-enzymatically treated feather meal), K6FM (K6 keratinase treated feather meal), K82FM (K82 keratinase treated feather meal), K6:K82FM [K6 and K82 keratinase (5:1) treated feather meal] and CMFM (commercial enzyme treated feather meal). The pepsin digestibility of CFM (70 %) and CMFM (68 %) was significantly higher than K6FM (60 %), K82FM (61 %) and K6:K82FM (63 %). Total amino acid content of K82FM (89.65/100 g) was the highest compared with the other treatments. The nutrient digestibility of the feather meals was determined for broiler chicks between 21 and 27 days old. The apparent nitrogen retention of K82FM (85.82 %) and K6FM (77.31 %) was significantly higher than K6:K82FM (55.42 %), CMFM (45.70 %) and CFM (48.16 %). The apparent metabolisable energy (AME_n_) was not significantly different between the feather meal treatments, although K82FM, K6FM and K6:K82FM showed AME_n_ higher than CMFM and CFM. The results indicated that both K6 and K82 keratinase had a positive effect on the protein quality of the feather meal produced by the enzymatic–hydrothermal method.

## Background

Feathers are produced in large amounts as by-product of poultry processing plant worldwide. The feather is 90 % keratin protein and the accumulation of feathers in the environment results in pollution and feather protein wastage (Belarmino et al. 2012; Gopinath et al. 2015; Gousterova et al. 2005; Onifade et al. 1998). Therefore, utilization of biological by-product as livestock feed is an accepted practice, which reduces costs both in terms of waste disposal and meat production from livestock (Grazziotin et al. 2006). Feathers have elevated keratin content and the use of this protein source should be considered. Traditional ways to degrade feathers such as alkali hydrolysis and steam pressure cooking not only destroy the amino acids, but also require large amounts of energy (Papadopoulos et al. 1986; Tiwary and Gupta 2012). The biodegradation of the feathers by keratinase from microorganisms could be a cost effective alternative. Keratinase and related products have many applications (Brandelli et al. 2010; Gupta and Ramnani 2006; Gupta et al. 2013). For example, the feather hydrolysates of *Bacillus licheniformis* PWD-1 and *Vibrio* sp. strain kr2 (Williams et al. 1991; Grazziotin et al. 2006) can be used as feed additives, while the keratinase from *Bacillus subtilis* S14 and *Brevibacillus brevis* US575 exhibits remarkable dehairing capabilities in the leather processing industry (Macedo et al. 2005; Jaouadi et al. 2013). Keratinase is very important for the utilisation of feather meal. The properties of keratinolysis are widespread in the microbial world. However, only few of these properties have been commercially exploited. Keratinases from *Bacillus* sp. particularly *B. licheniformis* and *B. subtilis* have been extensively studied for their efficiency in terms of feather degradation (Manczinger et al. 2003; Mazotto et al. 2011; Thys et al. 2004). A feather-degrading *B. licheniformis* KUB-K0006 and *B. pumilus* KUB-K0082 were discovered and isolated by Nitisinprasert and Kaewsompong (1997). These aerobic bacterial isolates possessed effective keratinase, with high feather digestibility at wide pH ranges and high temperatures of up to 50 °C. The enzyme keratinases were purified and characterised as a serine protease (Nitisinprasert et al. 1999; Titapoka 2003). Keratinases from *B. licheniformis* KUB-K0006 (K6 keratinase) and *B. pumilus* KUB-K0082 (K82 keratinase) displayed different abilities of feather digestion and quantities of amino acid released. K6 keratinase is endo-acting, whereas K82 keratinase is an exo-acting enzyme (Nitisinprasert et al. 1999). The aims of this study were to evaluate the effect of K6 and K82 keratinase on feather meal quality and nutrient digestibility at industrial scale production levels of broiler chicks.

## Results and discussion

### Keratinases preparation

**Fig. 1:**
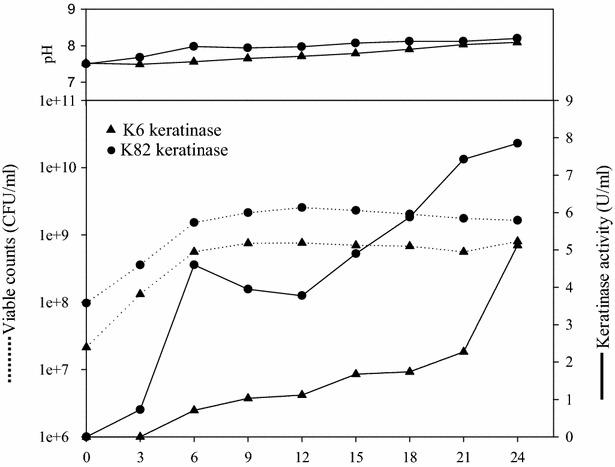
Production kinetics of K6 keratinase and K82 keratinase. All cultures were grown at 5 % inoculum, 37 °C for 24 h agitation speed 250 rpm and aeration rate 1.0 vvm

The keratinases production of *B. licheniformis* KUB-K0006 (K6 keratinase) and *B. pumilus* KUB-K0082 (K82 keratinase) were illustrated in Fig. 1. Fermentation was carried out with 5 % (v/v) inoculum, aeration rate at 1.0 l/min and without pH control (7.5 initial) at 250 rpm, 37 °C for 24 h in a 200 l fermentor. During the batch fermentation by both strains, the pH changed from 7.5 to 8.2 and showed similar growth characteristics. Both keratinases were produced in the late exponential or stationary phase of growth. *B. pumilus* KUB-K0082 produced higher keratinase activity (7.85 U/ml) than *B. licheniformis* KUB-K0006 (5.12 U/ml) at 24 h. The cell-free media were concentrated by membrane ultrafiltration with 30 kDa molecular weight cut off. The crude concentrated K6 and K82 keratinase had an activity of 33,054 and 53,295 U/l, respectively. In large-scale production, the keratinase activities from both strains were similar to the previous experiment (Nitisinprasert and Kaewsompong 1997; Nitisinprasert et al. 1999; Eaksuree et al. 2016). Scale-up effect was not observed in the production patterns. This revealed that both Bacilli were suitable for keratinase production in large scale.

### Characteristics of feather meals from industrial scale production

The proximate composition and pepsin digestibility of the five feather meal samples are shown in Table 1. The crude protein content of K82FM was significantly higher than the other treatments (*P* < 0.05). Both K6FM and K82FM showed significantly less pepsin digestibility than K6:K82FM, the control (CFM) and commercial enzyme (CMFM) (*P* < 0.05). Kim and Patterson (2000) reported the same result; the pepsin digestibility content of autoclaved enzyme feather meal was lower than autoclaved control feather meal. The pepsin digestibility of CFM was the highest but not significantly different from CMFM. There were no significant differences in the energy contents of CFM and CMFM and these two treatments showed significantly higher results than K6:K82FM, K6FM and K82FM (*P* < 0.05). The crude fat and the fat ball contents were significantly different in all experiments. The results indicated that K82FM was the best feather meal with the highest crude protein and lowest fat ball content.

**Table 1:** Proximate composition and pepsin digestibility of feather meal CFM, K6FM, K82FM, K6:K82FM and CMFM

Treatment	Fat ball (kg)	Crude fat (%)	Crude protein (%)	Ash (%)	Moisture (%)	Energy (Cal/100 g)	Pepsin digestibility (%)
CFM	204^a^	11.3^a^	81.4^c^	2.46^a^	2.14^d^	438^a^	70^a^
K6FM	186^c^	9.55^c^	80.6^c^	2.14^b^	5.38^a^	418^c^	60^c^
K82FM	115^e^	7.4^e^	88.6^a^	1.52^d^	1.25^e^	426^b^	61^c^
K6:K82FM (5:1)	192^b^	8.27^d^	84.9^b^	2.0^c^	3.11^b^	421^c^	63^b^
CMFM (commercial)	130^d^	11.5^b^	81.4^c^	1.95^c^	2.82^c^	438^a^	68^a^

CFM = non-enzymatically treated feather meal; K6FM = K6 keratinase treated feather meal; K82FM = K82 keratinase treated feather meal; K6:K82FM = K6and K82 keratinase (5:1) treated feather meal; and CMFM = commercial enzyme treated feather meal

^a,b,c,d,e^Means within a column with different superscripts differ significantly (P < 0.05)

The amino acid compositions of the feather meals are presented in Table 2. The amino acid content of the enzymatically treated feather meals ranged from 81.10 to 89.65 % all higher than the control CFM (78.31 %). The alanine, arginine, aspartic acid, cystine, glutamic acid, glycine, histidine, isoleucine, leucine, lysine, methionine, phenylalanine, proline, serine, threonine, tryptophan, tyrosine and valine levels of K82FM were significantly higher than K6FM, K6:K82FM and CMFM. The enzymatic hydrolysis of feather meals were observed to be rich in valine, leucine and lysine content, which were higher than feather meal from *B. licheniformis* ER-15 (Tiwary and Gupta 2012). However, the histidine, leucine, lysine, proline, tyrosine and valine contents of CMFM were significantly higher than the other treatments (*P* < 0.05). The results showed that keratinolytic enzyme treatment at high temperature and pressure could increase the amino acid content in the feather meal. This result agreed with Kim and Patterson (2000) and Tiwary and Gupta (2012), who reported that enzymatic treatment produced higher amino acid content in feather meal preparation.

**Table 2:** Amino acid composition of feather meal CFM, K6FM, K82FM, K6:K82FM and CMFM

Amino acid (g/100 g)	Treatment				
	CFM	K6FM	K82FM	K6:K82FM	CMFM
Alanine	3.78^a^	4.05^c^	4.43^e^	4.23^d^	4.01^b^
Arginine	5.61^a^	5.84^b^	6.34^e^	6.29^d^	6.06^c^
Aspartic acid	4.91^a^	4.95^b^	5.74^e^	5.46^d^	5.16^c^
Cystine	3.85^b^	4.03^c^	4.56^e^	4.19^d^	3.48^a^
Glutamic acid	8.95^a^	8.98^a^	10.26^d^	9.84^c^	9.74^b^
Glycine	6.83^a^	7.43^b^	7.92^e^	7.75^d^	7.54^c^
Histidine	0.82^a^	0.89^c^	0.84^ab^	0.86^bc^	0.89^c^
Hydroxylysine	–	–	–	–	–
Hydroxyproline	0.18^c^	0.15^bc^	0.11^a^	0.15^bc^	0.13^ab^
Isoleucine	3.35^b^	3.31^a^	3.61^c^	3.64^c^	3.93^d^
Leucine	6.18^a^	6.38^b^	7.12^d^	7.03^c^	7.24^e^
Lysine	1.91^a^	1.88^a^	1.95^b^	1.97^b^	2.03^c^
Methionine	0.56^a^	0.56^a^	0.61^b^	0.67^c^	0.56^a^
Phenylalanine	3.48^b^	3.51^b^	4.24^d^	3.37^a^	3.64^c^
Proline	8.46^a^	9.07^b^	9.91^d^	9.72^c^	10.27^e^
Serine	9.75^a^	10.10^b^	11.62^e^	11.30^d^	10.59^c^
Threonine	3.81^a^	3.94^b^	4.39^e^	4.31^d^	4.18^c^
Tryptophan	0.57^a^	0.70^b^	0.77^c^	0.77^c^	0.68^b^
Tyrosine	0.86^a^	0.92^bc^	0.90^b^	0.95^cd^	0.98^d^
Valine	4.45^b^	4.41^a^	4.78^c^	4.79^c^	5.14^d^
Total amino acids	78.31	81.10	89.65	87.29	86.25

CFM = non-enzymatically treated feather meal; K6FM = K6 keratinase treated feather meal; K82FM = K82 keratinase treated feather meal; K6:K82FM = K6and K82 keratinase (5:1) treated feather meal; and CMFM = commercial enzyme treated feather meal

^a,b,c,d,e^Means within a row with different superscripts differ significantly (*P* < 0.05)

### Effect of feather meal on nutrient digestion of broiler chicks

Five diets were formulated for the growing phase. CFM, K6FM, K82FM, K6:K82FM and CMFM were substituted for the corn soybean meal diet at levels of 10 %. The apparent nitrogen retention (ANR), apparent fat digestibility (AFD) and apparent metabolisable energy (AME_n_) are shown in Table 3. Data analysis showed that the ANR of Diet 2 (K6FM) and Diet 3 (K82FM) were significantly higher than Diet 4 (K6:K82FM), Diet 1 (CFM) and Diet 5 (CMFM). There was no significant difference in ANR between Diet 2 and Diet 3 or between Diet 4, Diet 1 and Diet 5 (*P* > 0.05). The diet containing 10 % K82FM showed the highest ANR among the treatments.

**Table 3:** Apparent nitrogen retention (ANR), apparent fat digestibility (AFD) and apparent metabolisable energy (AME_n_) in feed samples

Treatment diet	Apparent nitrogen retention (%)	Apparent fat digestibility (%)	Apparent metabolisable energy (N-corrected) (kcal/kg)
Diet 1 (control)	48.16^b^	63.05	3005.82
Diet 2 (K6FM)	77.31^a^	82.40	3277.05
Diet 3 (K82FM)	85.82^a^	88.20	3445.42
Diet 4 (K6:K82FM)	55.42^b^	72.92	3255.58
Diet 5 (CMFM)	45.70^b^	68.76	3148.77
P value	0.0009	0.9089	0.9793
Pool ± SE	11.4751	32.2577	949.0794

Diet 1 = corn soybean meal + 10 % CFM; Diet 2 = corn soybean meal + 10 % K6FM; Diet 3 = corn soybean meal + 10 % K82FM; Diet 4 = corn soybean meal + 10 % K6:K82FM; and Diet 5 = corn soybean meal + 10 % CMFM

^a,b^Means within a column with different superscripts differ significantly (*P* < 0.05)

There were no significant differences in the AFD and apparent metabolisable energy among the treatments (*P* > 0.05). However, Diet 3 (K82FM) showed the highest AME_n_, 440 kcal/kg higher than Diet 1 (CFM). This indicated that the feather meal prepared by keratinase at high temperature and pressure increased the metabolisable energy levels of the broilers. The results revealed that K82FM was a good alternative protein source, with high levels of AFD and AME_n_.

*In vivo* digestibility, metabolisable energy and protein (nitrogen) retention of the enzymatic treated feather meal showed an inverse relationship with in vitro pepsin digestibility. However, the amino acid content of the feather meal with enzyme treatments was higher than without the treatment (Table 2). Papadopoulos (1987) concluded that pepsin digestibility should not be considered in the evaluation of feather meal as a poultry feed. Although the trends in values were similar, there was poor correlation between in vitro and in vivo tests of protein quality, therefore care should be taken when comparing reports in which different methods of testing have been used.

## Conclusions

The feather meals prepared from hydrolysis reactions using concentrated crude K6 and K82 keratinase were tested for nutrient digestibility in the broiler chicks. Results showed higher ANR for K6FM and K82FM in vivo. Nevertheless, crude protein and total amino acid contents of K82FM were highest compared against the other feather meal treatments. The value of pepsin digestible protein (PDP) of the feather meal produced from the commercial enzyme (CMFM) was the highest, but K6FM and K82FM were found to improve the ANR of the chicks better than CMFM. There were no significant differences in apparent metabolisable energy among the feather meal treatments, however K6 and K82 keratinase feather meal treatments showed higher AME_n_ than the commercial enzyme feather meal treatment (180 kcal/kg). Therefore, the K6 and K82 keratinase showed potential use for industrial feather meal production.

## Methods

### Enzyme preparation

K6 keratinase and K82 keratinase were prepared from *B. licheniformis* KUB-K0006 and *B. pumilus* KUB-K0082, respectively. The fermentation medium contained (g/l) NH_4_Cl (0.5), NaCl (0.5), K_2_HPO_4_.3H_2_O (0.354), KH_2_PO_4_ (0.4), MgCl_2_·6H_2_O (0.24) and feather meal (10 % w/v), with initial pH at 7.5. The production was performed in a 200 l fermenter (FM-300A, B.E. Marubishi, Japan), aeration rate 1.0 l/min, agitation speed 250 rpm at a temperature of 37 °C for 24 h. Inoculation volume was 5 %. The cell-free media were concentrated by membrane ultrafiltration with 30 kDa molecular weight cut off (Sartorius model SM 17546, Germany). The activity of crude concentrated K6 and K82 keratinase was measured by the hydrolysis of milled feather (Lin et al. 1992).

### Feather meal production

Feather meals were prepared at industrial scale production (3.8 tonnes feather/batch). The concentrated crude K6 keratinase, K82 keratinase, K6:K82 (5:1) and commercial enzyme were used in the feather meal production. A total of 3.8 tonnes of native feathers were collected from the slaughter house of B. Foods Product International Co., Ltd. (Thailand) and then transferred into a cooker/drier with keratinase enzyme (493,600 U/batch). The feathers were incubated at 60 °C for 1 h and then autoclaved at 130 °C for 20 min. After hydrolysis, the feathers were dried to 10 % moisture content (maximum value) and hammer milled to obtain 3 mm particle size. The feather meal was stored in a silo. The samples were analysed; moisture (ISO 1999), crude fat (AOAC 2000), ash, protein, pepsin digestibility and amino acid profiles (AOAC 2005) at Betagro Science Center Co., Ltd. (Thailand).

### Diet

A corn soybean meal diet was formulated at 20 % crude protein, 8.25 % fat and 3.86 % fibre (Table 4). A corn soybean meal diet was formulated to provide similar nutrient profile according to the broiler’s nutrient as recommended by National Research Council (NRC, 1994). Five diets included CFM (non-enzymatically treated feather meal), K6FM (K6 keratinase treated feather meal), K82FM (K82 keratinase treated feather meal), K6:K82FM (K6 and K82 keratinase (5:1) treated feather meal) and CMFM (commercial enzyme treated feather meal). The feather meal from each treatment was added to replace corn soybean meal diet at 10 % (w/w). All diets were prepared in crumble form and contained chromic oxide (0.3 %) as a digestibility marker.

**Table 4:** Diet composition and nutrient content of corn soybean meal diet

Ingredient (g/kg)	Control diet
Corn	541.2
Soybean meal, 440 g/kg CP	358.0
Soybean oil	60.0
Mono-dicalcium phosphate (MCP)	17.3
Lime stone	14.5
dl-Methionine	2.3
Salt	4.2
Premix^a^ (vitamin and mineral)	2.5

Calculated composition	Amount
ME for poultry (kcal/kg)	3150.0
Crude protein	200.0
Ash	28.2
Crude fat	82.5
Crude fibre	38.6
Calcium	9.5
Phosphorus-available	4.2
Salt	5.0
Lysine	11.0
Methionine	5.4
Methionine + cysteine	8.5
Sodium	1.8
Choline (mg/kg)	1300.0

^a^Composition of Premix per kg of diet: vitamin A (retinol acetate), 2.7 mg; vitamin D_3_ (cholecalciferol), 0.125 mg; vitamin E (dl-tocopheryl acetate), 33 mg; vitamin K_3_ (menadione), 3 mg; vitamin B_1_ (thiamin), 2 mg; vitamin B_2_ (riboflavin), 6 mg; vitamin B_6_ (pyridoxine), 3 mg; vitamin B_12_ (cobalamin), 0.016 mg; nicotinic acid, 60 mg; pantothenic acid, 15 mg; niacin, 5.18 g; folic acid, 1.75 mg; biotin, 0.1 mg; Cu, 16 mg; Zn, 100 mg; Fe, 40 mg; Mn 120 mg; Se, 0.3 mg; I, 1.25 mg

### Chickens and management

The study protocol was approved by the Animal Ethics Committee of Kasetsart University, Thailand. One hundred and twenty male broiler chicks aged 21 days (Ross 308, Betagro Agro-Group Public Co. Ltd.), were randomly allocated to 20 metabolic cages. Each treatment was replicated four times with six birds per cage. The birds were housed in a room maintained at a controlled temperature of 25 °C with ventilation and lighting. Feed and water were provided to the chicks for ad libitum intake throughout the experiment. Test diets were provided for the 21 day old chicks when they were placed in the cages. After 4 days of experimental diet feeding (adjusting period), all the feeders were removed and all the excreta trays were cleaned. The amount of remaining feed was recorded and the feeders were then reinstalled. This was considered as the starting point of the feed intake and excreta collection period. During the 72 h collection period, excreta samples of 200 g for each replicate were collected once daily at 8:00 a.m. All excreta trays were cleaned at 4:00 pm in the evening to prevent contamination. The wet excreta was adjusted to pH 4.0 using 6 N sulphuric acid and then stored at −4 °C until required for testing. At the end of the collection period the samples were defrosted, homogenized, sun dried and 200 g aliquots were dried in an oven at 70 °C to constant weight. Dried excreta and feed samples were milled and analysed for moisture, gross energy (GE), crude protein and crude fat (AOAC 1990). Chromic oxide was measured following the method of Bolin et al. (1952). ANR, AFD and apparent metabolisable energy corrected for nitrogen (AMEn) were calculated according to the method of Hill et al. (1960).

### Statistical analysis

The data were subjected to analysis of variance (ANOVA) arranged in completely randomized design (CRD). Significant differences between treatment groups were detected by Duncan’s multiple range test and contrast comparison analysis (SAS, 2003). Means were compared and considered significant when *P* < 0.05.
